# Reply to: “Comment on the Role of Muscle Mass Gain Following Protein Supplementation Plus Exercise Therapy in Older Adults with Sarcopenia and Frailty Risks: A Systematic Review and Meta-Regression Analysis of Randomized Trials, *Nutrients* 2019, *11*, 1713”

**DOI:** 10.3390/nu11102420

**Published:** 2019-10-10

**Authors:** Chun-De Liao, Hung-Chou Chen, Shih-Wei Huang, Tsan-Hon Liou

**Affiliations:** 1 Department of Physical Medicine and Rehabilitation, Shuang Ho Hospital, Taipei Medical University, Taipei 23561, Taiwan; 08415@s.tmu.edu.tw (C.-D.L.); 10462@s.tmu.edu.tw (H.-C.C.); 13001@s.tmu.edu.tw (S.-W.H.); 2 School and Graduate Institute of Physical Therapy, College of Medicine, National Taiwan University, Taipei 10055, Taiwan; 3 Center for Evidence-Based Health Care, Shuang Ho Hospital, Taipei Medical University, Taipei 23561, Taiwan; 4 Graduate Institute of Sports Science, National Taiwan Sport University, Taoyuan 33371, Taiwan; 5 Department of Physical Medicine and Rehabilitation, School of Medicine, College of Medicine, Taipei Medical University, Taipei 11031, Taiwan

We thank Chen et al. [1] for their interest and comments on our recent publication [2]. We appreciate the authors’ comprehensive concerns bringing some obvious faults to our attention. Here are our responses to concerns that they raised.

First, the follow-up duration assessed in the included randomized control trial (RCT) was initially defined as follows: immediate follow up (<3 months), short-term follow up (≥3 months, <6 months), medium-term follow up (≥6 months, <12 months), and long-term follow up (≥12 months) [2]. In the present study [2], which included 19 RCTs for meta-analysis, only one RCT (Hegerová et al. [3]) assessed lean body mass (LBM) outcome at a long-term follow up of 12 months, no other included RCT reported long-term outcomes. Hegerová’s results were not fully indicated in Figure 2 of the current meta-analysis [2] and the results may be misunderstood as what has been commented by Chen [1]. We added the information regarding the long-term outcome of LBM in the Supplementary Figure S1 to clarify the effects of protein supplement (PS) combined with muscle strengthening exercise (MSE) on each follow-up time period; the additional result showed a significant long-term effect in favor of PS + MSE (standard mean difference (SMD) = 1.26; 95% CI: 0.95–1.56; *p* < 0.00001; Supplementary Figure S1) and the originally reported overall effect on LBM was not changed by this added result. We concluded that PS + MSE exerted benefits on LBM and appendicular lean mass (ALM) at short-term and medium-term follow up; in addition, PS + MSE had a long-term effect on LBM.

Secondly, the factors which influence the effects of PS + MSE on ALM were not clearly indicated in our recent meta-analysis [2]. We clarify the findings from the results of subgroup analyses for ALM as follows: a significant difference (*I*^2^ = 79.2%; *p* = 0.03) in the effect of PS + MSE on ALM was identified between RCTs with high (SMD 0.63; 95% CI 0.31, 0.95) and low (SMD 0.06; 95% CI −0.17, 0.29) methodological quality; additionally, a significant difference (*I*^2^ = 76.0%; *p* = 0.02) in the effect of PS + MSE on ALM was found among participant condition subgroups, which was in line with the result in LBM; no significant difference among all other subgroups was observed in terms of effects on ALM (Table 3 in the meta-analysis [2]).

Thirdly, the authors (Chen et al.) have concerns regarding the meta-regression results [1]. As suggested, we reperformed meta-regression analyses by removing the outliers indicated in Figures 3 and 4 in the published study [2], and adding an additional covariate of sex for multivariate regression models (Supplementary Table S1). The reanalyzed results showed that, after controlling for age, sex, methodological quality, and follow-up time, intervention-induced changes in ALM (in percentage) were significantly associated with the SMD of leg strength (*β* = 0.33, 95% CI 0.02–0.64; *p* < 0.05) and walk capability (*β* = 0.29, 95% CI 0.06–0.52; *p* < 0.05). In addition, we indicate that Figures 3–5 in the published meta-analysis [2] were plotted by RStudio software (RStudio, Inc., Boston, MA, USA [4]) using term.plot function of gamlss package in R [5].

Finally, Chen et al. pointed out an interesting finding that significant effects on muscle strength and walk capability were identified at short-term follow up, whereas at medium-term follow up they were not. The inconsistence of the results between short-term and medium-term follow up may be attributed to the different populations. At short-term follow up of handgrip, leg strength, and walk capacity, most comparisons (74–84%) were studied for sarcopenic older individuals, whereas all comparisons at medium-term follow up were investigated for frail older individuals. The subgroup analysis results in the published meta-analysis had shown that participants’ conditions had significant influence on leg strength (*I*^2^ = 88.7%, *p* = 0.0001; Table 3) [2]; sarcopenic older people may have achieved greater effects in leg strength (SMD = 0.73, *p* < 0.00001) in response to PS + MSE than did the frail peers (SMD = 0.58, *p* < 0.0001); similar results were observed in walk capability despite nonsignificant subgroup differences (*I*^2^ = 49.3%, *p* > 0.05). According to the results of the subgroup analysis for participants’ conditions, more evident or greater intervention effects may be observed at short-term follow up for muscle strength and walk capability, compared with those at medium-term follow up.

We hope this letter clarifies the points raised by Chen. We are appreciative of the opportunity to provide a corrected Figure 2 and to reply to these important questions.

## Supplementary Materials

The following are available online at https://www.mdpi.com/2072-6643/11/10/2420/s1, Figure S1: Forest plot summarizing effects of protein supplement (PS) plus muscle strengthening exercise (MSE) on changes of muscle mass, muscle strength, and physical function at each follow up duration. Table S1: Association of muscle mass changes (%) with effect size (SMD) of leg strength and walk capability.
