# Supplementary material for: Reply to: “Comment on the Role of Muscle Mass Gain Following Protein Supplementation Plus Exercise Therapy in Older Adults with Sarcopenia and Frailty Risks: A Systematic Review and Meta-Regression Analysis of Randomized Trials, Nutrients 2019, 11, 1713”
Source: Nutrients. 2019 Oct 10;11(10):2420. doi: 10.3390/nu11102420 (PMC6835344; doi:10.3390/nu11102420)
Supplement: Supplementary file 1 [file nutrients-11-02420-s001.pdf]

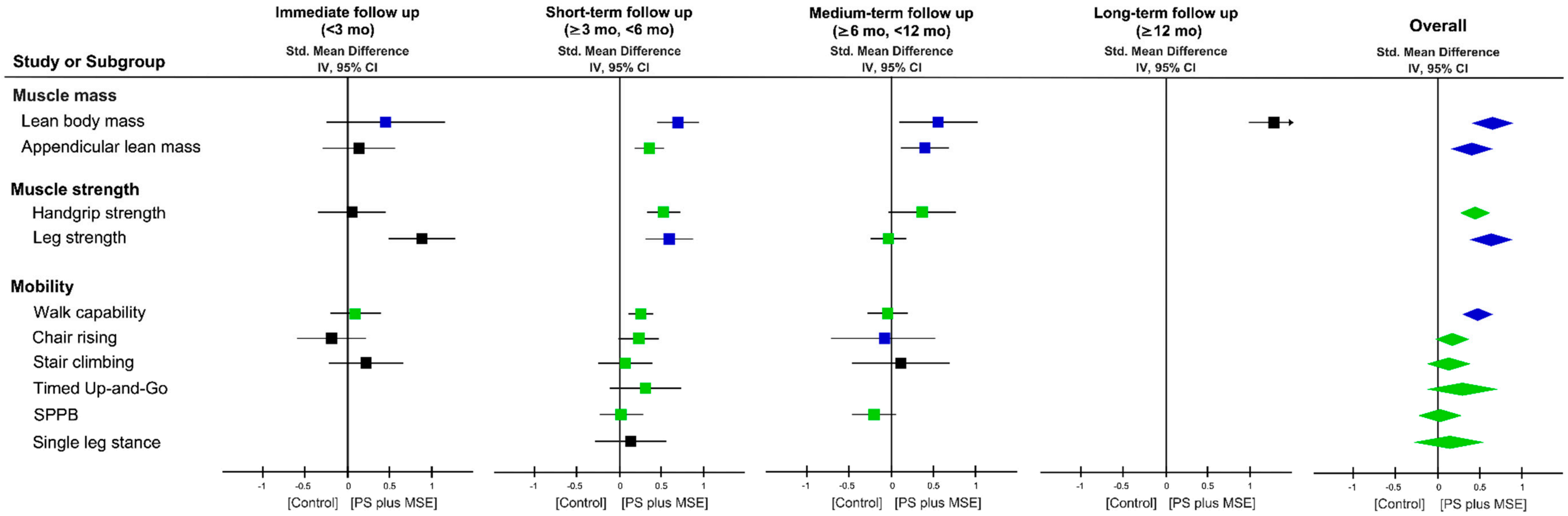

Figure S1. Forest plot summarizing effects of protein supplement (PS) plus muscle strengthening exercise (MSE) on changes of muscle mass, muscle strength, and physical function at each follow up duration. Each point estimate at each follow up duration (square) and during an overall duration (diamond) presents the combined effect (standard mean difference) of the outcome measure where indicated, with 95% CI (horizontal line). Results plotted on the right-hand side indicate effects in favor of PS plus MSE. The combined effects analyzed by a fixed- or random-effect model are denoted by green and blue colors, respectively; and a black colored square denotes that the combined effect is derived from a single study. 95% CI: 95% confidence interval; Std.: standard; IV: inverse variance; SPPB: short physical performance battery.

Table S1. Association of muscle mass changes (%) with effect size (SMD) of leg strength and walk capability.

Dependent Variable	Model 1 [a]				Model 2 [b]			
Covariate¶	Coefficient (β)	95% CI	R^2	p Value	Coefficient (β)	95% CI	R^2	p value
SMD of leg strength			0.45	0.004			0.78	0.004
LBM (change, %)	0.52	(0.19, 0.85) *			0.38	(−0.15, 0.91)		
Age (year)					−0.001	(−0.057, 0.055)		
Sex					−0.10	(−0.56, 0.37)		
MQ					0.40	(0.10, 0.71) *		
FU (week)					−0.01	(−0.10, 0.08)		
SMD of leg strength			0.40	0.01			0.86	0.002
ALM (change, %)	0.39	(0.09, 0.68) **			0.33	(0.02, 0.64) *		
Age (year)					−0.07	(−0.15, 0.01)		
Sex					−0.64	(−1.33, 0.05)		
MQ					0.49	(0.18, 0.80) **		
FU (week)					0.03	(−0.05, 0.13)		
SMD of walk capability			0.26	<0.001			0.96	<0.001
ALM (change, %)	0.58	(0.32, 0.84) ***			0.29	(0.06, 0.52) *		
Age (year)					0.03	(−0.01, 0.05)		
Sex					0.63	(0.29, 0.97) **		
MQ					0.17	(−0.05, 0.39)		
FU (week)					0.05	(−0.04, 0.14)		

* $p < 0.05$, ** $p < 0.01$, *** $p < 0.001$. All muscle mass variables were transformed to percentage change from baseline; MQ is classified as high and low with a PEDro (Physiotherapy Evidence Database) score ≥7/10 and <7/10, respectively; [a] Model 1: Univariate linear regression variables included muscle mass measures. [b] Model 2: Multivariate linear regression variables included age, sex (men, women, and mixed), MQ (high and low), follow-up time, and muscle mass measures from Model 1. Code for category variable: Sex (women = 1, mixed = 2, men = 3); MQ (low = 1, high = 2). SMD: standard mean difference; LBM: whole body lean mass; MQ: methodological quality; FU: follow-up time; ALM: appendicular lean mass.
